# Compound 49b Regulates ZO-1 and Occludin Levels in Human Retinal Endothelial Cells and in Mouse Retinal Vasculature

**DOI:** 10.1167/iovs.16-20412

**Published:** 2017-01

**Authors:** Youde Jiang, Li Liu, Jena J. Steinle

**Affiliations:** 1 Department of Anatomy and Cell Biology, Wayne State University School of Medicine, Detroit, Michigan, United States; 2 Department of Ophthalmology, Wayne State University School of Medicine, Detroit, Michigan, United States

**Keywords:** Epac1, Occludin; ZO-1, retinal endothelial cell, conditional knockout

## Abstract

**Purpose:**

To investigate whether Epac1 is key to Compound 49b's regulation of zonula occluden 1 (ZO-1) and occludin levels in human retinal endothelial cells (REC) and in an Epac1 vascular-specific conditional knockout mouse retina.

**Methods:**

Primary REC were grown in normal (5 mM) or high glucose (25 mM). Some cells were treated with a novel β-adrenergic receptor agonist, Compound 49b. Additional dishes were treated with Epac1 siRNA or Compound 49b + Epac1 siRNA. Protein levels of ZO-1, occludin, VEGF, and protein kinase C zeta (PKCz) were measured by Western blotting. Cell permeability was measured in REC grown in normal or high glucose and treated with Compound 49b, a specific Epac 1 agonist (8-CPT-2′-O-Me-cAMP), or VEGF. Epac1 floxed and cdh5-Cre mice were bred to generate Epac1 knockout mice in vascular endothelial cells. Immunofluorescence was done on retinal flatmounts from the floxed and Cre-Lox mice for occludin and ZO-1. Western blotting was also done for both proteins in whole retinal lysates from the mice.

**Results:**

High glucose significantly reduced ZO-1 and occludin protein levels, which was associated with reduced cell adhesion. Compound 49b increased endothelial cell barrier protein levels through active Epac1. Knockout of Epac1 in vascular endothelial cells substantially reduced ZO-1 and occludin staining in retinal flatmounts, as well as protein levels.

**Conclusions:**

Compound 49b increased ZO-1 and occludin protein levels, likely leading to decreased permeability.

Diabetic retinopathy is the vision threatening complication of both type 1 and 2 diabetes. Statistics suggest that approximately 60% of patients will develop some component of retinopathy within 20 years of diagnosis.^1^ One of the common complications of diabetic retinopathy is diabetic macular edema. While anti-VEGF therapy is effective for a subset of these patients, there is a population of diabetic patients that do not respond adequately to anti-VEGF treatment. We recently reported that a novel β-adrenergic receptor agonist, Compound 49b, could significantly reduce VEGF levels through activation of protein kinase C (PKC) zeta in retinal endothelial cells (REC).^2^ Because others have reported that PKCzeta inhibitors can blunt VEGF-induced dysfunction of retinal barriers,^3^ we questioned whether Compound 49b could regulate retinal barrier proteins and cellular adhesion.

Because macular edema is a critical complication of retinopathy, much work has been done to investigate the blood–retinal barrier (BRB) in diabetes. Diabetes is associated with increased VEGF levels, which contribute to increased permeability.^4^ The BRB is formed by the retinal pigmented epithelium and blood vessels of the retina to form the outer and inner barriers, respectively. The control of fluids and solutes across the BRB is regulated by tight junctions. A key family of tight junction proteins in the retina is zonula occludens (ZO). For this study, we have focused on ZO-1, which was the first ZO identified.^4^ ZO-1 can physically bind the distal C-terminus of occludin, a transmembrane tight junction protein reported to be key to BRB maintenance.^4,5^

Because occludin can be regulated by VEGF and we know that Compound 49b can inhibit VEGF, we sought to find signaling partners downstream of β-adrenergic receptors that may could regulate both VEGF and occludin. Work in human umbilical cord blood endothelial cells showed that Epac1 significantly reduced permeability in these cells.^6^ Epac1 typically will activate Rap1.^7^ Others have also reported that both Epac1 and PKA can regulate human umbilical vein endothelial cells (HUVEC) barrier properties through parallel and independent signaling pathways.^8^

We have already shown that Compound 49b could reduce VEGF,^2^ as well as activate PKA,^9^ thus the goal of this study was to investigate whether active Epac1 was required for Compound 49b to regulate REC barrier proteins. We hypothesized that Compound 49b would be unable to increase ZO-1 and occludin when Epac1 was knocked down by siRNA. Furthermore, ZO-1 and occludin levels will be reduced in the vasculature of Epac1 vascular-specific conditional knockout mice.

## Methods

### Retinal Endothelial Cell Culture

Primary human REC were acquired from Cell Systems Corporation (CSC; Kirkland, WA, USA). Cells were grown in Cell Systems medium supplemented with microvascular growth factors, 10 μg/mL gentamycin, and 0.25 μg/mL amphotericin B (Invitrogen, Carlsbad, CA, USA) on attachment factor coated dishes. Once cells reached confluence, some dishes were moved to Cell Systems Medium with supplemented D-glucose to 25 mM. Only cells prior to passage 6 were used. Cells were quiesced by incubating in high or normal glucose medium without microvascular growth supplement (MVGS) for 24 hours prior to experimental use.

### Treatments

Retinal endothelial cells in normal (5 mM) and high glucose (25 mM) were transfected with Epac1 siRNA (SMARTpool: ON-TARGETplus RAPGEF3 siRNA; Dharmacon, Lafayette, CO, USA) at a final concentration of 20 nM using RNAiMAX transfection reagent according to the manufacturer's instructions. Twenty-four hours after transfection, cells in each condition were treated with 50 nM Compound 49b for an additional 24 hours. Analyses were done on REC samples after 3 to 4 days in normal or high glucose and 24 hours after Compound 49b treatment.

### Permeability Assay

Retinal endothelial cells were grown in normal and high glucose medium in transwell chambers. Once cells were 100% and treated with hydrocortisone, some cells in each condition were treated with 8-CPT-2′-O-Me-cAMP, a specific Epac1 agonist. In additional cells, 50 nM Compound 49b or 10 μM VEGF was added to the cells for 24 hours. On the day of the experiments, 70 kD rhodamine isothiocyanate (RITC) dextran was added to the upper chamber of the transwell insert. Aliquots from the basolateral chamber were taken every 30 minutes for 2 hours and placed into the 96-well plate. At 2 hours (the last time-point), a sample was also taken from the apical chamber to insure fluorescence in this chamber was unchanged. All aliquots were placed into a 96-well black plate for measurement using a fluorescent plate reader (SynergyHT; Biotek Instruments, Winnoski, VT, USA) at 570/590. The diffusive flux (Po) was calculated as reported by.^10,11^

### Mice

All animal procedures meet the ARVO Statement for the Use of Animals in Ophthalmic and Vision Research and were approved by the Institutional Animal Care and Use Committee of Wayne State University (Detroit, MI, USA) and conform to National Institutes of Health guidelines. The Epac1 floxed mice (B6;129S2-Rapgef3^tm1Geno/J^ mice) and B6 FVB-Tg (cdh5-cre)7Mlia/J Cre mice were purchased from Jackson Laboratories (Bar Harbor, ME, USA). After 2 generations, the Epac1 floxed mice were bred with the cdh5-Cre mice to generate conditional knockout mice in which Epac1 is eliminated in vascular endothelial cells. At 3 months of age, Epac1 floxed and Epac1 Cre-Lox mice were used for experiments.

### Genotyping

Genomic DNA was extracted from ear punch of 3-week-old mice. Tissues were digested with one step tail DNA extract buffer (100 mM Tris, 5 mM EDTA, 200 mM NaCL, 1% Triton) plus proteinase K (10 mg/mL) at 55°C overnight, followed by enzyme heat-inactivation at 85°C for 45 minutes. Sequences of primer pairs used to screen the Epac1 conditional knock out mice were as follows: Epac1: 5′->3′ mutant forward: ATT TGT CAC GTC CTG CAC GAC G, wild type forward: CTG GCC TCT CCT GAA TCT TG, common: CCT CGC TGT TGG TAA GTG GT. Cdh5-cre forward: AGG CAG CTC ACA AAG GAA CAA T; reverse: TCG TTG CAT CGA CCG GTA A; Cdh5-cre internal positive control forward: CTA GGC CAC AGA ATT GAA AGA TCT; reverse: GTA GGT GGA AAT TCT AGC ATC C. The standard PCR reaction was done using KAPA2G HotStart Genotyping PCR Mix (KK5621; KAPA Biosystems, Wilmington, MA, USA). Polymerase chain reaction was performed with following temperatures and times: denature: 95°C, 3 minutes; 35 cycles at 95°C, 15 seconds; 60°C, 15 seconds; 72°C sec/kb; with final extension at 72°C, 1 minute. Confirmation of mouse genotyping results is in a paper currently under review (Liu et al., 2016).

### Immunofluorescence

Four mice from Epac1 Cre-lox and corresponding control floxed mice were used for immunofluorescence of ZO-1 and occludin. Mice were euthanized under the CO2, followed by cervical dislocation. The eye cup was enucleated and placed into 4% paraformaldehyde for 2 hours. After rinsing in PBS, the retina was gently dissected out. For detecting tight junction proteins, retinal flatmounts were incubated in 5% BSA with 0.3% Triton for 2 hours, followed by ZO-1 (rat polyclonal, 1:250; EMD Millipore, Bilerica, MA, USA) or occludin (rabbit polyclonal, 1:500; Novus Biologicals, Littleton, CO, USA) and isolectin GS-IB4, which was conjugated with Alexa Fluor 488 (1:100; Life Technologies, Invitrogen, Carlsbad, CA, USA) for 2 days at 4°C. After rinsing in PBS with 0.3% Triton, retinal flatmounts were transferred to secondary antibody (goat anti rat Alexa Fluor 555 or goat anti rabbit Alexa Fluor 594 (1:500; Life Technologies) overnight at 4°C. After rinsing in PBS, the retinas were flat mounted onto slides and examined under a Leica Confocal microscope (Buffalo Grove, IL, USA).

Additional mice were used for Western blot protein analyses.

### Western Blotting

After appropriate treatments and rinsing with cold PBS, REC or whole retinal lysates were collected into lysis buffer containing protease and phosphatase inhibitors. Equal amounts of protein from the cell extracts were separated on the precast tris-glycine gel (Invitrogen), blotted onto a nitrocellulose membrane. After blocking in TBST (10 mM Tris-HCl buffer, pH 8.0, 150 mM NaCl, 0.1% Tween 20) and 5% (wt/vol) BSA, the membranes was treated with Epac1, PKCzeta, Occludin, ZO-1, VEGF (all from Abcam, San Francisco, CA, USA), or beta actin (Santa Cruz Biotechnology, Santa Cruz, CA, USA) primary antibodies followed by incubation with secondary antibodies labeled with horseradish peroxidase. Antigen-antibody complexes were detected by chemilluminescence reagent kit (Thermo Scientific, Pittsburgh, PA, USA). Data was acquired using an Azure C500 (Azure Biosystems, Dublin, CA, USA). Western blot analyses were done using Image Studio Light software (LI-COR, Lincoln, NE, USA).

### Statistical Analyses

Nonparametric Kruskal-Wallis with Dunn's post hoc tests were used for the cell culture data. *P* less than 0.05 was considered statistically significant. A representative blot is shown for Western blot data.

## Results

### Epac1 is Key to Compound 49b Actions on REC Permeability

**Figure 1:**
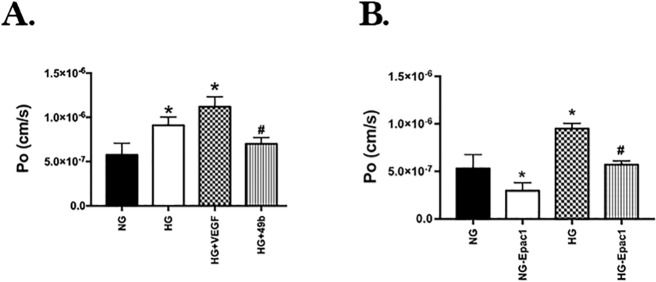
Compound 49b requires Epac1 to decrease permeability. *Panel A* is a bar graph of the diffusive flux on REC grown in normal glucose (NG) or high glucose (HG) or HG + 50 nM Compound 49b or 10 μM VEGF. *N* = 4 for each group. *P < 0.05 versus NG, #*P* < 0.05 versus HG. *Panel B* shows the flux measurements of REC grown in normal or high glucose and treated with an Epac1 specific agonist. *N* = 4 for each group. *P < 0.05 versus NG, #*P* < 0.05 versus HG.

We performed calculations of diffusive flux as a measurement of permeability on REC grown in normal or high glucose and treated with an Epac1 agonist. Figure 1A shows that high glucose significantly increased RITC-dextran flux in the REC, which was also slightly increased further by VEGF treatment. Compound 49b decreased the flux in REC grown in high glucose. Figure 1B shows that high glucose culturing conditions significantly increased the flux in the REC, which was blocked by the addition of the Epac1 agonist. The data in Figure 1 suggest that Compound 49b can decrease flux through Epac1 actions.

### Compound 49b Requires Active Epac1 to Reduce PKCzeta and VEGF

**Figure 2:**
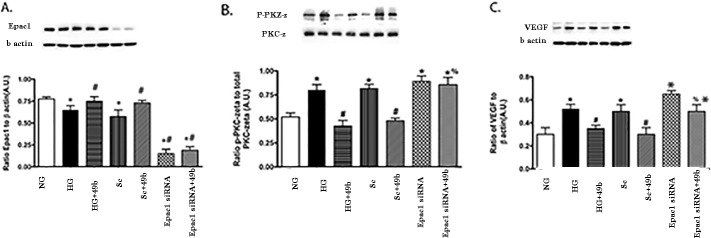
Epac1 depletion increases VEGF expression and PKCzeta activation. Retinal endothelial cells were grown in NG, HG, HG + Compound 49b, HG + scrambled siRNA (HG + Sc), HG + Compound49b + scrambled siRNA (Sc + 49b), HG + Epac1 siRNA, or HG + Compound 49b + Epac1 siRNA (Epac1 siRNA + 49b). *Panel A* is a control to show Epac1 knockdown. *Panel B* is the ratio of phosphorylated PKCzeta to total PKCzeta. *Panel C* is VEGF levels compared with beta actin. *N* = 4 for each group of cells. *P < 0.05 versus NG, #*P* < 0.05 versus HG, %*P* < 0.05 versus HG + Compound 49b. A representative blot is provided for each group.

Because we have previously reported that Compound 49b could reduce VEGF through activation of PKCzeta, we wanted to ascertain whether Epac1 was key to this regulation. Figure 2 demonstrates that high glucose significantly increases both PKCzeta and VEGF, which was blocked by Compound 49b, as we reported in the past.^2^ Compound 49b was unable to reduce PKCzeta (B) and VEGF (C) when Epac1 levels are reduced via Epac1 siRNA. Figure 2A shows that Epac1 siRNA was effective in reducing Epac1 levels.

### Compound 49b Cannot Increase ZO-1 When Epac1 is Blocked

**Figure 3:**
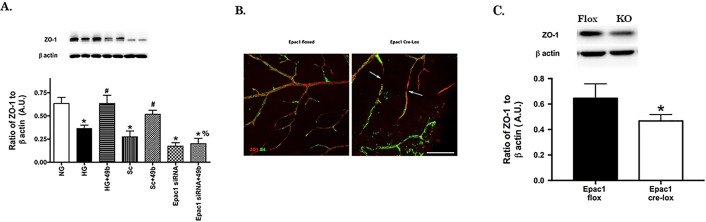
ZO-1 levels are not increased by Compound 49b when Epac1 is knocked down. *Panel A* shows ZO-1 levels in REC grown in NG, HG, HG + Compound 49b, HG + scrambled siRNA (HG + Sc), HG + Compound49b + scrambled siRNA (Sc + 49b), HG + Epac1 siRNA, or HG + Compound 49b + Epac1 siRNA (Epac1 siRNA + 49b). *N* = 4 for each group of cells. *P < 0.05 versus NG, #*P* < 0.05 versus HG, %*P* < 0.05 versus HG + Compound 49b. A representative blot is provided for each group. *Panel B* shows immunostaining of ZO-1 in Epac1 floxed mouse retina versus cdh5/Epac1 Cre-Lox mice retina. *Scale bar* is 50 μm. Red staining is ZO-1 and Isolectin B4 is *green. Arrows* point to gaps along the vessel with no ZO-1 staining. Staining is representative of four mice in each group. *Panel C* provides Western blot results for ZO-1 levels in the Epac1 floxed and conditional knockout mice. *N* = 4, *P < 0.05 versus floxed.

ZO-1 protein levels are significantly reduced in REC grown in high glucose. This can be reversed with Compound 49b treatment (Fig. 3A). However, Compound 49b cannot increase ZO-1 in REC transfected with Epac1 siRNA, suggesting that Epac1 is key to regulation of ZO-1 in REC. Similarly, mouse retina from Epac1 floxed mice (left) have substantially higher ZO-1 staining than retinal sections from cdh5/Epac1 Cre-Lox mice, where Epac1 is eliminated in vascular endothelial cells (Fig. 3B). The Epac1 conditional knockout mice also have significant gaps in ZO-1 staining (arrows). Figure 3B is a representative image of four mice in each group analyzed. Figure 3C shows that ZO-1 protein levels are significantly increased in the Epac1 floxed mice when compared with Epac1 Cre-Lox. This data suggests that Epac1 may play a key role in regulating ZO-1 in the retina. Supplementary Figure S1 shows a representative image of ZO-1 staining from all four mice.

### Epac1 is Critical to Compound 49b's Actions on Occludin

**Figure 4:**
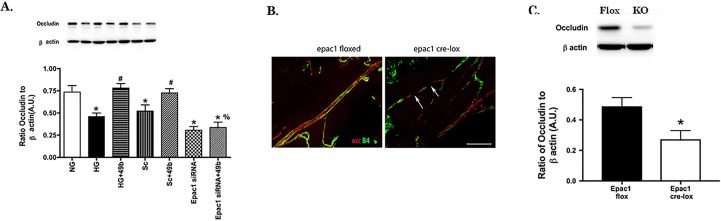
Occludin is regulated by Epac1. *Panel A* shows occludin levels in REC grown in NG, HG, HG + Compound 49b, HG + scrambled siRNA (HG + Sc), HG + Compound49b + scrambled siRNA (Sc + 49b), HG + Epac1 siRNA, or HG + Compound 49b + Epac1 siRNA (Epac1 siRNA + 49b). *N* = 4 for each group of cells. *P < 0.05 versus NG, #*P* < 0.05 versus HG, %*P* < 0.05 versus HG + Compound 49b. A representative blot is provided for each group. *Panel B* shows representative immunostaining of occludin in Epac1 floxed mouse retina versus cdh5/Epac1 Cre-Lox mice retina. *Red* staining is occludin and Isolectin B4 is green. *Arrows* point to gaps along the vessel with no occludin staining. Staining is representative of four mice in each group. *Panel C* provides Western blot results for occludin levels in the Epac1 floxed and Cre-Lox mice. *N* = 4, *P < 0.05 versus floxed.

Because ZO-1 and occludin often interact to maintain the retinal barrier, we also studied the effects of Epac1 knockdown on occludin levels. Retinal endothelial cells grown in high glucose had significantly reduced levels of occludin. Compound 49b was able to increase occludin levels considerably (Fig. 4A). However, Compound 49b was not able to increase occludin levels in REC transfected with Epac1 siRNA. Loss of Epac1 in the vascular endothelial cells of the mouse retina led to substantially lower occludin levels when compared with Epac1 floxed retina (Fig. 4B). Figure 4B is a representative image of four mice in each group analyzed. The staining is supported in Figure 4C showing increased occludin in the Epac1 floxed mice when compared with the conditional knockout mice. Taken together, these data strongly suggest that maintenance of Epac1 signaling may be key to regulating specific barrier proteins in the retina. Supplementary Figure S1 shows a representative image of occludin staining from all four mice.

## Discussion

Maintenance of the BRB is key to retinal health. Dysfunction of these barriers occurs relatively early in retinopathy progression, likely leading to a plethora of other pathologic events. Increasing our understanding of the regulation of key barrier proteins may offer new targets for retinal health. Loss of barrier protein actions can lead to diabetic macular edema, as well as provide the initiating steps for retinal detachment. We recently reported that Compound 49b, a novel β-adrenergic receptor agonist, significantly reduced VEGF levels in REC through inhibition of PKCzeta.^2^ Because β-adrenergic receptor agonists can work through multiple signaling cascades, the goal of these studies was to investigate whether Compound 49b could regulate REC barrier proteins and the role of Epac1 in this regulation. Using diffusive flux as a measurement of cell permeability, data show that a specific Epac1 agonist reduced the high glucose-induced increase in flux. Thus, it appears that Epac1 is involved in the maintenance of REC barrier properties in vitro. We focused on Epac1 as a downstream mediator of Compound 49b actions, as both PKA and Epac1 are reported to regulate endothelial cell barrier functions.^8^ While it is probable that both PKA and Epac1 may function in barrier actions, work in HUVEC cells showed that Epac1 had a regulatory role in cell junctions through VE-cadherin.^12^ As we have already demonstrated that Compound 49b activates PKA to protect REC from apoptosis,^9^ we focused on alternative downstream factors that may regulate specific actions common in diabetic retinopathy.

An additional reason that we focused specifically on Epac1 for these studies was a report demonstrating that human umbilical cord endothelial cells have increased permeability with age. This age-induced increase in permeability was substantially reduced in cells with elevated Epac1 levels.^6^ Additionally, increased permeability of the cells was correlated with tyrosine phosphorylation of occludin with age.^6^ Additional support of a role for Epac1 actions on endothelial cell junctional proteins was recently provided when Epac1 knockout mice demonstrated increased permeability in all organs studied, as well as less interendothelial junctional complexes.^13^ The work in Epac1 knockout mice is supported by other work in HUVEC cells showing that the herbal extract WS1442 increased Epac1, leading to Rac1-cortical actin rearrangement to reduce permeability.^14^ Furthermore, Epac1 activation reversed microtubule-dependent increases in permeability induced by TNFα in HUVECs.^15^ Thus, it appears in macrovascular endothelial cells, activation of Epac1 can regulate barrier proteins and decrease permeability. With our focus on the retina, we generated vascular endothelial cell–specific Epac1 knockout mice to investigate key barrier proteins in the retinal. Retinal samples from cdh5/Epac1 Cre-Lox mice demonstrated reduced occludin and ZO-1 immunostaining and protein levels when compared with the Epac1 floxed mice, suggesting that loss of Epac1 decreased endothelial cell barrier proteins. In support of the data from mice, REC treated with Compound 49b following Epac1 siRNA transfection showed that Compound 49b was unable to significantly increase ZO-1 or occludin without active Epac1. Thus, both the animal data and cell culture work strongly suggest that Epac1 can regulate occludin and ZO-1 in the retinal vasculature, likely leading to decreased permeability in vitro.

Taken together, these data suggest that Epac1 is key to Compound 49b's ability to regulate REC barrier protein levels. Because systemic administration of a β-adrenergic receptor agonist will likely not be feasible due to cardiovascular effects, these data suggest that increased Epac1 actions in the diabetic retina may prevent barrier protein dysfunction in response to high glucose. Future work will explore Epac1 actions in diabetic-like conditions.

## Supplementary Material

Supplementary Figure S1

## References

[1] American Diabetes Association. Eye Complications (2013) Available at: http://www.diabetes.org/living-with-diabetes/complications/eye-complications/.

[2] Jiang Y, Zhang Q, Steinle JJ. Beta-adrenergic receptor agonist decreases VEGF levels through altered eNOS and PKC signaling in diabetic retina. *Growth Factors*. 2015; 33: 192–199. 26115368 10.3109/08977194.2015.1054990 PMC4791949

[3] Titchenell PM, Lin CM, Keil JM, Novel atypical PKC inhibitors prevent vascular endothelial growth factor-induced blood-retinal barrier dysfunction. *Biochem J*. 2012; 446: 455–467. 22721706 10.1042/BJ20111961 PMC3767384

[4] Erickson KK, Sundstrom JM, Antonetti DA. Vascular permeability in ocular disease and the role of tight junctions. *Angiogenesis*. 2007; 10: 103–117. 17340211 10.1007/s10456-007-9067-z

[5] Harhaj NS, Felinski EA, Wolpert EB, VEGF activation of protein kinase C stimulates occludin phosphorylation and contributes to endothelial permeability. *Invest Ophthalmol Vis Sci*. 2006; 47: 5106–5115. 17065532 10.1167/iovs.06-0322

[6] Cheung TM, Ganatra MP, Peters EB, Truskey GA. Effect of cellular senescence on the albumin permeability of blood-derived endothelial cells. *Am J Physiol Heart Circ Physiol*. 2012; 303: H1374–H1383. 23023872 10.1152/ajpheart.00182.2012 PMC3532541

[7] Gloerich M, Bos JL. Epac: defining a new mechanism for cAMP action. *Annu Rev Pharmacol Toxicol*. 2010; 50: 355–375. 20055708 10.1146/annurev.pharmtox.010909.105714

[8] Lorenowicz MJ, Fernandez-Borja M, Kooistra MR, Bos JL, Hordijk PL. PKA and Epac1 regulate endothelial integrity and migration through parallel and independent pathways. *Eur J Cell Biol*. 2008; 87: 779–792. 18635287 10.1016/j.ejcb.2008.05.004

[9] Zhang Q, Steinle JJ. DNA-PK phosphorylation of IGFBP-3 is required to prevent apoptosis in retinal endothelial cells cultured in high glucose. *Invest Ophthalmol Vis Sci*. 2013; 54: 3052–3057. 23557743 10.1167/iovs.12-11533 PMC3640744

[10] Antonetti DA, Wolpert EB, DeMaio L, Harhaj NS, Scaduto RC Jr. Hydrocortisone decreases retinal endothelial cell water and solute flux coincident with increased content and decreased phosphorylation of occludin. *J Neurochem*. 2002; 80: 667–677. 11841574 10.1046/j.0022-3042.2001.00740.x

[11] Chang YS, Munn LL, Hillsley MV, Effect of vascular endothelial growth factor on cultured endothelial cell monolayer transport properties. *Microvasc Res*. 2000; 59: 265–277. 10684732 10.1006/mvre.1999.2225

[12] Kooistra MR, Corada M, Dejana E, Bos JL. Epac1 regulates integrity of endothelial cell junctions through VE-cadherin. *FEBS Lett*. 2005; 579: 4966–4972. 16115630 10.1016/j.febslet.2005.07.080

[13] Kopperud RK, Rygh CB, Karlsen TV, Increased microvascular permeability in mice lacking Epac1 (RapGef3) [published online ahead of print April 20, 2016] *Acta Physiol (Oxf)*. doi: http://dx.doi.org/10.1111/apha.12697. 10.1111/apha.12697 PMC5073050 27096875

[14] Bubik MF, Willer EA, Bihari P, A novel approach to prevent endothelial hyperpermeability: the Crataegus extract WS(R) 1442 targets the cAMP/Rap1 pathway. *J Mol Cell Cardiol*. 2012; 52: 196–205. 22085704 10.1016/j.yjmcc.2011.10.020

[15] Sehrawat S, Cullere X, Patel S, Italiano J Jr, Mayadas TN. Role of Epac1, an exchange factor for Rap GTPases, in endothelial microtubule dynamics and barrier function. *Mol Biol Cell*. 2008; 19: 1261–1270. 18172027 10.1091/mbc.E06-10-0972 PMC2262967

