# Supplementary material for: Compound 49b Regulates ZO-1 and Occludin Levels in Human Retinal Endothelial Cells and in Mouse Retinal Vasculature
Source: Invest Ophthalmol Vis Sci. 2017 Jan;58(1):185–9. doi: 10.1167/iovs.16-20412 (PMC5256679; doi:10.1167/iovs.16-20412)
Supplement: Supplementary Figure S1 [file iovs-57-15-30_s01.pdf]

Supplemental Figure 1

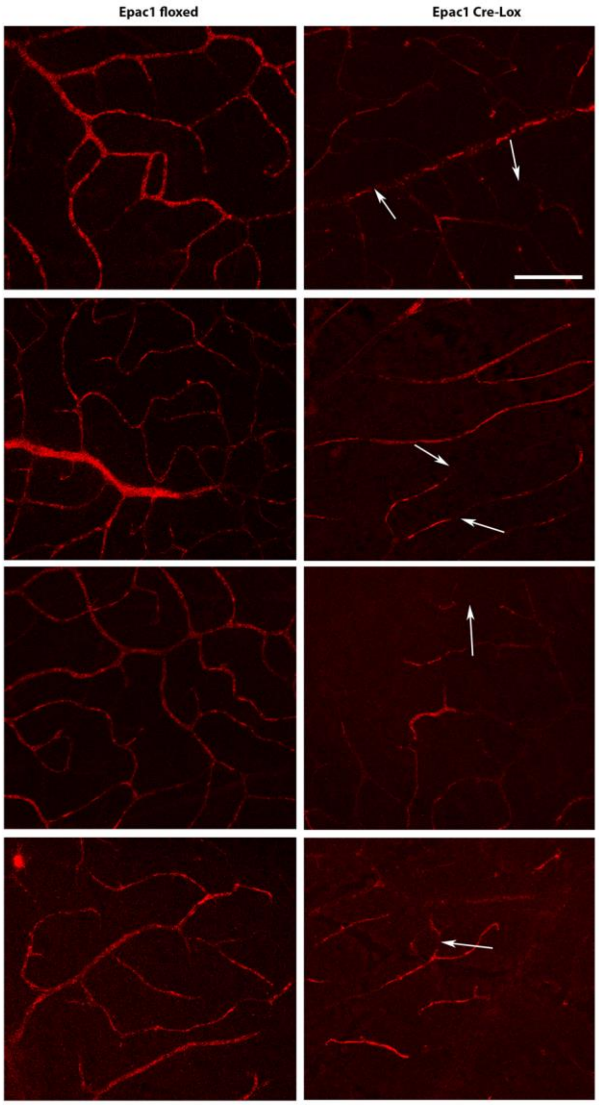

Supplementary Figure 1. Representative ZO-1 immunostaining from all 4 Epac1 floxed and Epac1 Cre-Lox mice. Scale bar is 50um. Arrows show locations of gaps in staining.

Supplemental Figure 2

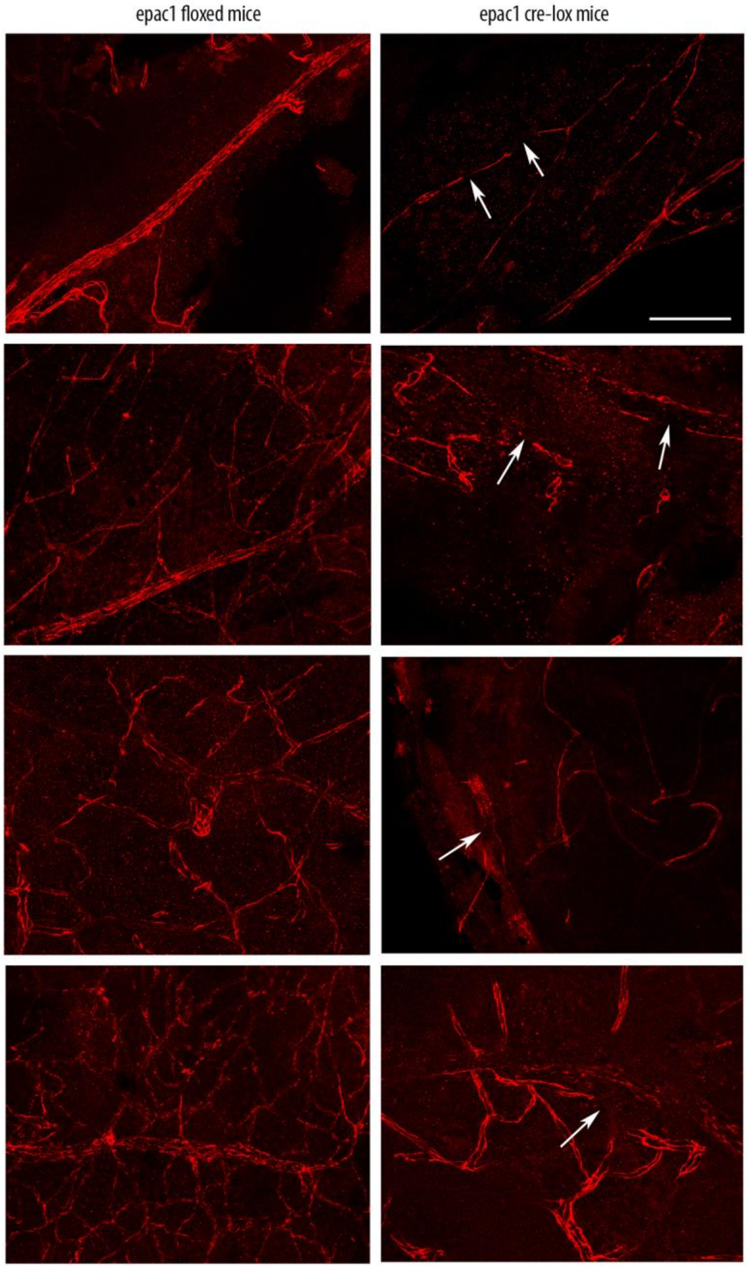

Supplementary Figure 2. Representative occludin immunostaining from all 4 Epac1 floxed and Epac1 Cre-Lox mice. Scale bar is 50um. Arrows show locations of gaps in staining.
